# Should young people be paid for getting tested? A national comparative study to evaluate patient financial incentives for chlamydia screening

**DOI:** 10.1186/1471-2458-12-261

**Published:** 2012-04-02

**Authors:** Dominik Zenner, Darko Molinar, Tom Nichols, Johanna Riha, Mary Macintosh, Anthony Nardone

**Affiliations:** 1 Health Protection Agency, Centre for Infections, London, UK; 2 National Chlamydia Screening Programme, Health Protection Agency, Centre for Infections, London, UK

**Keywords:** Chlamydia, Screening, Incentives, Observational study, Sexual health

## Abstract

**Background:**

Patient financial incentives ("incentives") have been widely used to promote chlamydia screening uptake amongst 15-24 year olds in England, but there is scarce evidence of their effectiveness. The objectives of the study were to describe incentives used to promote chlamydia screening in Primary Care Trusts (PCTs) in England and to evaluate their impact on coverage and positivity rate.

**Methods:**

PCTs that had used incentives between 1/1/2007 and 30/6/2009 (exposed) were matched by socio-demographic profile and initial screening coverage with PCTs that had not (unexposed). For each PCT, percentage point change in chlamydia screening coverage and positivity for the period before and during the incentive was calculated. Differences in average change of coverage and positivity rate between exposed and unexposed PCTs were compared using linear regression to adjust for matching and potential confounders.

**Results:**

Incentives had a significant effect in increasing average coverage in exposed PCTs (0.43%, CI 0.04%-0.82%). The effect for voucher schemes (2.35%) was larger than for prize draws (0.16%). The difference was greater in females (0.73%) than males (0.14%). The effect on positivity rates was not significant (0.07%, CI -1.53% to 1.67%).

**Conclusions:**

Vouchers, but not prize draws, led to a small absolute but large relative increase in chlamydia screening coverage. Incentives increased coverage more in females than males but had no impact on reported positivity rates. These findings support recommendations not to use prize draws to promote chlamydia screening and contribute to the evidence base of the operational effectiveness of using patient incentives in encouraging public health action.

## Background

*Chlamydia trachomatis *is the most common sexually transmitted infection (STI) in England and the overall prevalence is estimated to be about 5% in the general population under 20 years of age [1]. The National Chlamydia Screening Programme (NCSP) was established in 2003 to target all 15-24 year olds in England to help prevent its potentially serious health complications [2] and to reduce onward transmission.

Patient financial incentives ("PFIs") have been used for a range of intended health behaviour changes [3,4] and there is some evidence of effect in simple behaviour changes such as attending a meeting. However, they were found much less effective to achieve more complex and longer term changes in behaviour [4]. Incentives have been widely used to increase chlamydia screening coverage in England even though there is scarce evidence of their effect on screening coverage or the rate of chlamydia positivity [4-6]. There is also no evidence on how these schemes may affect the age and sex composition of the population accepting screening.

The aim of this study was to describe the financial initiatives schemes in England and to evaluate their impact among 15-24 year olds by comparing chlamydia screening coverage and positivity rate changes in Primary Care Trusts (PCTs) that had or had not used incentives. We also compared the effect different schemes had on the demographic characteristics of those accepting screening.

## Methods

### Definition of patient financial incentives

Financial incentives were defined as any goods (monetary or non-monetary) exchanged with a patient against the desired action (chlamydia screen). The acquisition of goods not depending on the desired action (e.g. handing out tokens during outreach events) was excluded. PFI schemes had to cover the majority of the PCT area.

All PFI schemes between 1^st ^January 2007 and 30^th ^June 2009 were described (n = 65). The analysis of these schemes was limited to prize draw and voucher schemes (n = 46) that could be matched (n = 42) with PCTs that had not used these schemes. Incentives based on tokens with items of negligible monetary value (e.g. sweets, condoms, panties, n = 19) were excluded from this analysis due to large variations in how these schemes operated.

### Data sources

The Central Office of Information (COI) carried out a national survey of PCTs' use of PFI and health promotion activities in April 2009 (response rate 75%). Information from this survey was used to identify PCTs which had or had not used incentives.

Telephone interviews with PCT screening leads were conducted in September and October 2009 using a semi-structured questionnaire to gather detailed information on any incentive schemes and major health promotion activities including mass mail-outs carried out during the period of interest. We also confirmed that all schemes had had safeguards against double-testing or providing inappropriate samples. We interviewed screening leads from all 46 PCTs which used incentives (as identified by the COI survey) as well as PCTs where inadequate or incomplete information was available from this survey. Of the 84 PCTs included in the matched analysis, 73 (87%) were interviewed. Eleven unexposed PCTs were not interviewed as the information from the COI survey was deemed to be of sufficient quality. All PCTs selected for telephone interviews were successfully contacted. We further validated information on the incentive schemes and other relevant health promotion campaigns using routinely collected data from the NCSP.

#### Chlamydia screening coverage and positivity rates

Screening coverage and positivity were calculated using routine data collection from the NCSP. For each chlamydia screen reported to the NCSP there is additional information on test result, and socio-demographic data. These data are collated centrally by the NCSP and used to ascertain the numbers and demographic characteristics of those screened.

Chlamydia testing coverage rates per quarter for 15-24 year olds were calculated from the number of screens among residents of a PCT using 2007 mid-year population estimates from the Office of National Statistics (ONS) as the denominator. Positivity was calculated as the number of positive screens per total screens in a quarter.

Our analysis was restricted to opportunistic screening; persons tested for clinical reasons (4.8% of the data submitted to the NCSP) were excluded. Screens performed in prisons or military settings (4%) were also excluded. Equivocal, inhibitory or insufficient test results (2.6%) were excluded from the positivity analysis. Data on exposure, outcome and potential confounders was complete. Screens returned with missing age or sex were excluded from all analyses (complete case analysis).

### Statistical analysis

For each PCT, we calculated the percentage point change in chlamydia screening coverage and positivity in the period before and during the incentive. We estimated the difference in average change of the coverage and the positivity rate between PCTs that had (exposed) and had not used incentives (unexposed). The analysis examined the effect on coverage and positivity over two consecutive quarters: the quarter during which the PCT started to use a PFI, and the quarter immediately prior to the introduction of the PFI. Time periods used to calculate changes were always the same within a matched pair of PCTs, but varied between pairs.

PCTs which had employed incentives were matched to unexposed PCTs by choosing the ones with the most similar screening coverage in the quarter prior to the PFI from the same ONS health area supergroup (clusters of socio-demographically similar areas) [7]. A 1:1 matching was performed; PCTs which used more than one PFI could be matched more than once if schemes were more than six months apart. The PCT-pairing was kept for all analyses. Percentage point changes in screening and positivity rates were analysed at PCT level.

A single variable analysis was performed. We differenced the percentage point change of an exposed and unexposed pair and then averaged these differences. The analysis of changes in coverage and positivity were performed in the same fashion, using paired t-tests.

Multivariable analysis used linear regression on observations for each PCT, and potential confounders were considered and included if they were thought to affect the comparison between exposed and unexposed PCTs. We investigated the potential difference in the effect of prize draws and voucher incentives by using an exposure variable with 3 categories ("prize draw", "voucher" and "no incentive"). Stratification of the data by age and sex was to investigate the potential difference between the effect on females compared to males, and the effect on 15-19 year olds compared to 20-24 year olds. We divided the data for each PCT between four observations - one for each combination of sex and age group. We always allowed for the pairing by controlling for a variable that identifies each PCT-pair.

All analyses were performed using *MS Excel XP *and *Stata *11 (Stata Corporation, College Station, TX). Differences in absolute change from baseline are presented as recommended by the Cochrane guidelines [8].

## Results

### Description of patient incentive schemes

Forty-six of the 152 PCTs in England had used a total of 65 PFI schemes to increase chlamydia screening coverage between January 2007 and June 2009. Twenty-nine PCTs had used such a scheme once, 15 twice and two three times. The majority of schemes (62%; 40/65) were prize draws, which offered rewards ranging in value from a £50 voucher to a £2000 holiday for four (Table 1). The remaining schemes used either tokens (29%; 19/65) or vouchers (9%; 6/65), which ranged in value from £5 to £10. Most incentives were delivered through outreach work (55%); with smaller proportions using postal deliveries (25%), clinical services (9%) or other means (11%). The use of PFI schemes by PCTs in England has increased from an average of 2.5 schemes per quarter in 2007 to 13 per quarter in the first six months of 2009 (Table 1).

**Table 1:** Description of patient financial incentive schemes used by PCTs to promote chlamydia testing, January 2007-June 2009 (n = 65)

Overview of patient financial incentives		
Prize draws	40	61.5%
*Wii*	*17*	*42.5%*
*i-Pod*	*8*	*20.0%*
*Shopping voucher*	*5*	*12.5%*
*Holiday*	*6*	*15.0%*
*Other prizes*	*4*	*10.0%*
Vouchers	6	9.2%
*value £5-9*		*33.3%*
*value £10*	*4*	*66.7%*
Tokens	19	29.2%
**Primary setting of administering scheme**		
***postal***	**16**	**24.6%**
*prize draw*	*13*	*81.3%*
*voucher*	*2*	*12.5%*
*tokens*	*1*	*6.3%*
***outreach***	**37**	**56.9%**
*prize draw*	*21*	*56.8%*
*voucher*	*1*	*2.7%*
*tokens*	*15*	*40.5%*
***clinical services***	**6**	**9.2%**
*prize draw*	*2*	*33.3%*
*voucher*	*1*	*16.7%*
*tokens*	*3*	*50.0%*
***other***	**6**	**9.2%**

**Period of schemes**		
*2007*	*10*	*15.4%*
*2008*	*29*	*44.6%*
*2009*	*26*	*40.0%*

### Characteristics of matched PCTs

Forty-two of the 46 prize-draw and voucher schemes were matched to unexposed PCTs for coverage and ONS supergroup. We excluded four schemes from the analysis because it was not possible to identify suitable matches from the same ONS supergroup and time. The demographic characteristics (e.g. population size or index of multiple deprivation, IMD) and NCSP activity (e.g. screening rates in the quarters before the incentive and service activities) were very similar in the exposed and unexposed PCTs (Table 2).

**Table 2:** Characteristics of matched PCTs (n = 42 pairs)

Exposed PCTs		Unexposed PCTs	P value
**eligible PCT population**			
average	41,267	46,883	0.38
smallest	15,600	18,200	
largest	86,000	152,000	
Ave. proportion of females 15-19	24.2%	24.1%	0.93
Ave. proportion of females 20-24	24.4%	24.5%	0.93
Ave. proportion of males 15-19	25.6%	25.6%	0.94
Ave. proportion of males 20-24	25.8%	25.8%	0.95
IMD* rank (of 152 PCTs)			
average	80	76	0.61
worst	7	6	
best	130	149	
**Average screens in the quarter before incentive**			
Number	954	1,040	0.63
Range	11 - 2793	22 - 4497	
Rate (per population of 15-24 olds)	2.37%	2.29%	0.77
**Year screening programme established**			
2003-2005	15	9	
2005-2007	5	18	
2007-2008	22	15	0.01
**Number of PCTs with mail-outs**			
Any mail-out 2007-2009	15	15	1.00
Mail-outs during incentive	11	8	0.43

*IMD (index of multiple deprivations) is a composite area deprivation index commonly used by the Office of National Statistics and other authorities in the UK. It currently consists of seven domains, measuring income, employment, health, education, environment, crime and housing in a small area

P values were calculated for differences between exposed and unexposed PCTs using t-test for continuous variables and χ^2 ^tests for proportions

### Difference in screening coverage

**Figure 1:**
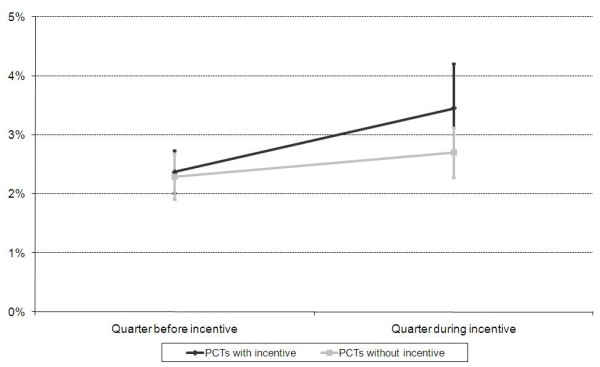
**Average Chlamydia screening coverage in exposed and unexposed PCTs in the quarter before and during the incentive time**. Bars depict 95% confidence intervals In the multivariable analysis we adjusted for the deprivation of the PCT (IMD quintile) (Table 3). Adjusting for these variables, the average screening rate change was 0.43% greater in PCTs which had used incentives compared to those that had not (*p *= 0.03).

Screening coverage increased in both exposed and unexposed PCTs (Figure 1) and in all 84 PCTs the average coverage in the quarter before a PFI was 2.3%. A single variable analysis demonstrated that unadjusted rate changes were more pronounced in PCTs which used incentives (1.08%, CI 0.48% to 1.68%) compared to those that had not (0.41%, CI 0.003% to 0.82%, Figure 1). The average difference between paired PCTs (0.67%, CI 0.1% to 1.24%) was significant (paired t-test *p *= 0.02).

The screening rate difference was more pronounced in voucher schemes compared to prize draws (2.35% vs. 0.16%), and in females compared to males (0.73% vs. 0.14%). Further analysis of voucher schemes found significant effects in females (3.18%) and to a lesser extent in males (1.55%).

Further analysis considered the predominant setting where the PFI was offered.

For prize draws we did not find any evidence for effect modification by setting in the regression model (*p *= 0.15). Whilst based on a small sample (n = 6), there was some evidence for effect modification for voucher schemes by setting (*p *= 0.02), but not for the value of the vouchers (*p *= 0.36, Table 3).

**Table 3:** Linear regression model of the difference in average percentage point change in screening coverage between exposed and unexposed PCTs

	Number of PCT Pairs	Difference in average % change	95% Conf. interval	P value	P value (Effect modif.)
Any financial incentive	42	0.43%	(0.04; 0.82)	0.03	
Males	42	0.14%	(-0.29%; 0.57%)	0.5	
Females	42	0.73%	(0.30%; 1.17%)	0.001	0.002
Prize draw	36	0.16%	(-0.22%; 0.54%)	0.4	
Vouchers	6	2.35%	(1.55%; 3.14%)	<0.0001	<0.0001
Prize draw in males	36	-0.05%	(-0.47%; 0.38%)	0.8	
Prize draw in females	36	0.37%	(-0.06%; 0.80%)	0.09	0.03
Vouchers in males	6	1.55%	(0.63%; 2.46%)	0.001	
Vouchers in females	6	3.18%	(2.25%; 4.11%)	<0.0001	0.0005
Prize draw via outreach	19	0.14%	(-0.40%; 0.68%)	0.606	
Prize draw via post	11	0.16%	(-0.39%; 0.71%)	0.57	
Prize draw via clinic	2	1.58%	(0.24%; 2.92%)	0.021	
Prize draw via other	4	0.03%	(-0.99%; 1.05%)	0.95	0.15
Voucher via outreach	1	3.15%	(1.11%; 5.20%)	0.003	
Voucher via post	2	3.66%	(2.24%; 5.08%)	<0.0001	
Voucher via clinic	1	2.24%	(0.25%; 4.23%)	0.027	
Voucher via other	2	1.10%	(-0.13%; 2.34%)	0.08	0.02
£5-9 Voucher	2	-0.66%	(-2.22%; 0.9%)	0.4	
£10 Voucher	4	2.65%	(1.70%; 3.59%)	<0.0001	0.36

The above is from a number of different models of screening coverage used in the analysis. The results are adjusted for the IMD quintile of the PCT and the pairing of the analysis. P Values in the first column are a test of whether the effect of the PFI is different from zero. P Values in the second column (effect modification) are a test of whether the effect of the PFI varies by the type of PFI (or by gender). Both significance tests are Wald tests.

We explored but found no evidence for effect modification by age group or by the length of time an incentive was run in a quarter affected by the PFI (1, 2 or 3 months). There was also no significant effect of health promotion campaigns or "simultaneous mail-out" (whether or not a mail-out was undertaken to promote an incentive).

All differences are between the average of PCTs with a particular sort of incentive and the average of PCTs without an incentive.

We found no evidence for effect modification by age group (15-19 or 20-24, *p *= 0.81), or number of months that the PFI was operational in a quarter (*p *= 0.5). We found no significant effect of major health promotion campaigns (*p *= 0.3) or mail-shot campaigns during the period of the PFI (*p *= 0.4) on differences in coverage rates.

### Differences in positivity rates

In all 84 PCTs the average positivity before a PFI was 7.24%. Overall there was little change in positivity between observed quarters (0.12% CI -0.52% to 0.76%). Employing the same matched pairs, the positivity rate change was 0.26% (CI -0.69% to 1.20%) and -0.01% (CI -0.91% to 0.89%) for unexposed and exposed PCTs respectively.

Adjusting for IMD quintile, the average difference in percentage point change between exposed and unexposed PCTs was small (0.07%, CI -1.53% to 1.67%) and not significant (*p *= 0.9). We found no evidence for effect modification by the type of incentive scheme (*p *= 0.7). Differences in positivity changes were 0.17% (CI -1.52% to 1.87%) and -0.55% (CI -4.33% to 3.23%) for prize draw and voucher schemes respectively.

We found no evidence for effect modification by age (*p *= 0.7) or sex (*p *= 0.5). The average difference in percentage point change was -0.08% (CI -1.96% to 1.8%, *p *= 0.9) and 0.22% (CI -1.65% to 2.09%, *p *= 0.8) for 16-19 and 20-24 years respectively. The average difference in percentage point change was -0.3% (CI -2.1% to 1.6%, *p *= 0.8) and 0.4% (CI -1.5% to 2.3%, *p *= 0.6) for males and females respectively.

## Discussion

We present the results of a national comparative study on the effect of incentives on chlamydia screening coverage and positivity rates. We found that voucher schemes significantly increased screening coverage and effects were more pronounced in females, but independent of age. Prize draw schemes did not significantly increase coverage in our study. The study did not find any evidence of an effect on positivity rates, either overall or by age, sex or type of scheme, and this could imply that the schemes do not lead to a substantial self-selection of lesser risk individuals.

The only randomised control trial, conducted in a single GP practice, did not observe any significant effect of vouchers on screening coverage [6]. Conversely, a recent quasi-experimental study amongst tertiary students in Australia found an effect of cash incentives on screening rates [9]; but intervention arms were not clearly defined and independent. Our findings of a significant effect of voucher schemes is based on observational evidence from a few schemes (n = 6). The larger overall effect sizes of voucher schemes could reflect a higher perceived value of vouchers compared to a prize draws, in keeping with the literature [10-12]. Incentives using higher value vouchers seemed to increase screening coverage, although not statistically significantly (*p *= 0.4). However, the majority (86%) of incentives evaluated in this study were prize draws and no significant impact was demonstrated for these.

A significant decline in chlamydia prevalence can only be achieved with high screening coverage and partner management [13] and cost-effectiveness depends on screening rates [14]. National policy aims are to increase screening coverage to 35-50% per year in the medium term [15]. The study covered the early period of the NCSP (2007-2009) when many PCTs were establishing local programmes and coverage achieved up to 12.5% in our study PCTs, similar to national coverage [14]. The observed 0.43% overall increase in coverage reported by those PCTs using incentives represents an 18% relative change and an average of 177 more screens per quarter, ranging from 80 more screens in prized draws to 600 in schemes using vouchers. These increases may have appeared attractive in the early stages of the programme. However, recent coverage rates are much higher (25% in October-December 2010) [14] and such numbers may appear less important in achieving high coverage in the population and it is possible that incentives will have lesser impact at higher coverage rates.

Although some studies have shown that incentives can be effective [3], currently NCSP does not recommend incentives outside research arrangements [16], based on a lack of evidence of their effectiveness [5,6] and concerns around sustainability and ethics. The majority of incentives in this study were conducted in non-clinical settings such as outreach, and these settings have lower positivity rates [10,17]. In the current climate of financial austerity, incentives are unlikely to provide a sustainable and cost effective addition to the strategic direction of focussing on core community services and aligning screening with health promotion [15]. In addition, whilst probably not coercive, incentives may have other unintended consequences, such as undermining patient-doctor relationships and patients' choices, while sustainability is debatable and controversy exists around the ethics of paying patients to adopt healthy behaviours amongst healthcare professionals [3,11,12,18] and the public [19].

Across all age-strata, there is evidence of a moderate effect of financial incentives on simple behaviours, such as attending meetings [20], but little impact on more complex and sustained behaviour changes like smoking cessation or weight loss [21,22]. The use of incentives in sexual health interventions has resulted in increased participation rates [22-25], but failed to improve sexual health outcomes [22,26]. However, incentives may have a role in overcoming barriers (e.g. norms and attitudes) to chlamydia screening in young people (aged 15-24) [27-29]. To achieve high levels of screening coverage will require complex rather than simple behaviour changes. Our findings of a small absolute overall effect is broadly consistent with the literature on complex behaviour changes [4].

The results from this observational study have evaluated efficiently and inexpensively the impact of all schemes during a 2 1/2 year period in England. Although we believe that bias and confounding is minimal, our observational findings will need to be confirmed in a trial. The number of voucher schemes was limited by their natural occurrence and may be too small to give conclusive results. The study was based on analyses of the NCSP database which contains all NCSP screening returns, but not those performed by other providers (i.e. those not registered and reporting to the NCSP). However, almost all incentives were coordinated by PCTs and/ or local NCSP screening coordinators who maintained the record for screening and reward. The relevance of these non-NCSP screens in this context is therefore probably small.

Misclassification of the PCTs with respect to the use of incentives is possible, but we believe unlikely. Information on financial incentive schemes and possible confounders such as other major PCT level activities was collected through a national marketing survey conducted by the COI. It is possible that some health promotion activities or mail-outs may have been missed in this study. However, this information was validated not only by telephone interviews with all PCTs who reported using incentives as well as the majority of control PCTs, but also with other readily available data from the NCSP. If exposure misclassification did occur, it would be most likely to be the misclassification of an exposed PCT as an unexposed PCT, leading to an underestimation of the effect of the incentive.

## Conclusions

In conclusion, we present the first national study of the effectiveness of incentives in chlamydia screening. The observational results should however be confirmed with a trial. Incentives have been used in a wide variety of contexts and our study adds to the discussion about their effectiveness beyond the immediate NCSP context. Based on our findings, prize draws are ineffective to increase screening uptake and further research needs to establish whether vouchers may play a role.

## Competing interests

The authors declare that they have no competing interests.

## Authors' contributions

DZ undertook the analysis and wrote the paper with contributions from DM, TN, JR, MM and AN. AN conceived the idea and TN contributed to and supervised the statistical analysis. AN supervised the study and the manuscript. All authors read and approved the final manuscript.

## Ethics approval

This study did not require formal ethics approval. Surveillance data collection had been approved to accompany and support the National Chlamydia Screening Programme (NCSP) in England, no additional approval was required to use this data for NCSP purposes (e.g. this study). The survey to evaluate financial incentive schemes was carried out amongst health care professionals only, and no patients have been approached.

## Data availability

The screening data is held by the NCSP. The survey used a tailored data collection amongst service leads. These data are not publicly available. Denominator calculation was performed using data from the Office of National Statistics in England, this data can be found on their website and is hence publicly available

## Funding

Internal HPA funding

## Pre-publication history

The pre-publication history for this paper can be accessed here:

http://www.biomedcentral.com/1471-2458/12/261/prepub
